# Evaluation of a Head-Mounted Virtual Reality Perimeter Compared with Stationary Perimeters across All Stages of Glaucoma

**DOI:** 10.1016/j.xops.2026.101314

**Published:** 2026-07-04

**Authors:** Marta Colmenar Herrera, Chen Hsin-Yang, Nathanael Häner, Serife S. Kucur Ergünay, Raphael Sznitman, Martin S. Zinkernagel

**Affiliations:** 1 ARTORG Center for Biomedical Engineering Research, University of Bern, Bern, Switzerland; 2 Graduate School for Cellular and Biomedical Sciences, University of Bern, Bern, Switzerland; 3 Department of Ophthalmology, Inselspital, Bern, Switzerland; 4 PeriVision SA, Epalinges, Switzerland

**Keywords:** Glaucoma, Visual fields, Virtual reality, Perimetry

## Abstract

**Objective:**

To evaluate agreement between a head-mounted virtual reality perimeter (VRP) and a conventional standard automated perimeter (SAP) in individuals with glaucoma across all stages of disease severity, including advanced visual field (VF) loss.

**Design:**

Prospective, single-center observational study.

**Participants:**

Adults with glaucoma undergoing VF testing; 120 matched VFs from 73 participants met the inclusion criteria.

**Methods:**

Participants underwent VF testing using a VRP (VisionOne; PeriVision AG) and SAP (Octopus 900; Haag-Streit AG) during a single visit. Global indices (mean sensitivity [MS], mean defect [MD]), pointwise sensitivity, and total deviation (TD) were compared. Agreement and association were assessed using Pearson correlation and Bland–Altman analyses. Stage-wise analyses evaluated interdevice differences across mild, moderate, and severe glaucoma. Test duration, reliability indices, and simulator-related discomfort were also assessed.

**Main Outcome Measures:**

Interdevice agreement for global VF indices (MS and MD), pointwise sensitivity and TD, and test performance measures (duration, reliability indices, and simulator-related discomfort).

**Results:**

Strong correlations were observed between VRP and SAP for global indices (MS and MD: ρ = 0.97; *P* < 0.0001) and for pointwise sensitivity and TD (sensitivity: ρ = 0.88; TD: ρ = 0.89; both *P* < 0.0001). Bland–Altman analyses demonstrated a small systematic pointwise sensitivity bias of –2.33 dB (95% limits of agreement [LoA]: –10.70 to +6.03 dB) and a TD bias of 0.98 dB (95% LoA: –6.36 to +8.33 dB), falling within known SAP test–retest variability parameters. Interdevice agreement remained consistent across glaucoma stages, including advanced VF loss. Test duration and reliability indices were comparable across devices. Simulator-related discomfort was generally low, with no test discontinuations.

**Conclusions:**

The VisionOne head-mounted VRP demonstrated strong agreement with conventional SAP for VF assessment, including cases of significant VF loss, supporting its clinical validity.

**Financial Disclosure(s):**

Proprietary or commercial disclosure may be found in the Footnotes and Disclosures at the end of this article.

Glaucoma remains one of the leading causes of irreversible blindness worldwide. Progressive loss of retinal ganglion cells results in characteristic visual field (VF) defects. Standard automated perimetry (SAP) remains the cornerstone for diagnosing and monitoring glaucoma. Conventional perimeters, such as the Octopus and Humphrey Field Analyzer systems, are bulky, require a controlled lighting environment and trained operators, and demand that users maintain a fixed posture and stable fixation for a prolonged period.^1^ These factors can cause discomfort and limit the accessibility and compliance of SAP, particularly in community screening or home-monitoring settings.^2^

Head-mounted virtual reality perimetry (VRP) integrates high-resolution displays and eye-tracking technology into a portable platform capable of reproducing standardized VF testing procedures in an immersive environment. Previous comparability studies have reported generally encouraging results, suggesting that VRP measurements can achieve clinically acceptable agreement with SAP under certain conditions.^3^ Nevertheless, differences in display luminance, dynamic range, background illumination, and fixation monitoring strategies remain technical factors that require explicit characterization and control.^3^^,^^4^ In our previous study published in 2021,^5^ a customized VRP system demonstrated noninferiority to the Octopus 900 in both healthy and glaucoma subjects, with good accuracy in detecting mild-to-moderate VF loss. More recent investigations have similarly compared VRP systems with conventional SAP, reporting strong correlations in global indices and pointwise sensitivities in healthy eyes and in subjects with mild-to-moderate glaucoma under controlled conditions.^3^ However, these studies have predominantly focused on intermediate disease stages and have not systematically evaluated performance in eyes with advanced VF loss, in part due to the luminance limitations of current VR display technologies. As a result, the accuracy, repeatability, and clinical reliability of VRP across the full spectrum of glaucomatous damage, and particularly in severe VF loss, remain incompletely characterized. While these findings provided important proof of concept, translation to routine clinical practice requires validation of commercially available, regulatory-approved VRP devices under standardized testing conditions and across all stages of disease severity.

Despite promising early results, the clinical adoption of VRP remains limited. Questions persist regarding the equivalence of global and pointwise sensitivity measures, test reliability, and patient tolerance when VRP is compared directly with established SAP devices. Many prior studies have focused on prototype systems or have used heterogeneous test parameters, making it difficult to isolate the effect of the testing platform itself.^3^ Consequently, systematic evaluations of clinically certified VRP systems using matched test strategies and outcome measures are needed to determine whether VRP can provide results comparable to those of SAP in glaucoma subjects, including those with advanced disease. Additionally, although immersive displays may offer ergonomic advantages, it is essential to assess their impact on user comfort during perimetric testing, as visual discomfort or fatigue associated with VR has been widely documented,^6^ but research focused on VRP remains limited.^3^

The primary objective of this study was to compare the global indices, pointwise sensitivity values, and reliability parameters obtained using a commercially available, regulatory-approved VRP system and a conventional SAP device in individuals with glaucoma. The secondary objective was to evaluate user experience by examining simulator-related discomfort using the Simulator Sickness Questionnaire, test duration, and reliability indices, thereby providing complementary insights into the clinical feasibility of VRP in routine glaucoma assessment.

## Methods

### Study Design

We conducted a prospective, single-center observational study at the Department of Ophthalmology, Inselspital, Bern University Hospital, Switzerland, between January and November 2025. Visual field testing was performed using a head-mounted VRP system (VisionOne™ [VO]; PeriVision AG) and a conventional SAP device (Octopus 900; Haag-Streit AG).

The sample size for the trial was calculated to evaluate differences across 4 patient age groupings. Using an analysis of variance model to account for group variances, a total sample size of 126 eyes was determined necessary to achieve 80% power with an effect size of 0.03 (Cohen f) and α=0.05. This manuscript reports a preplanned analysis of interdevice agreement utilizing the currently enrolled cohort of 73 participants (120 eyes). This sample size is sufficiently robust for the agreement analyses and aligns with or exceeds those of comparable perimetric validation studies.^3^

All study data were anonymized before analysis. The study protocol (identifier: 2024-01961) was approved by the Bernese Ethics Committee and adhered to the tenets of the Declaration of Helsinki. Written informed consent was obtained from all subjects before participation. Data are available from the corresponding author upon reasonable request.

### Participants

A total of 73 individuals with glaucoma were enrolled. Eligible participants were adults (≥18 years) with a confirmed diagnosis of glaucoma, refractive error within ±5 diopters (spherical equivalent), astigmatism <3 diopters, and best-corrected visual acuity better than 0.3 logMAR (0.5 decimal). Participants with prior experience with SAP were included. Exclusion criteria comprised inability to comply with test procedures, insufficient proficiency in the study language, coexisting ocular diseases (e.g., cataract), neurological conditions affecting the VF, and a history of epilepsy. To avoid introducing systematic bias toward interdevice agreement, favoring participants who performed well on both platforms, participants were not excluded based on asymmetric reliability performance across devices.

Participants were recruited during routine glaucoma clinic visits after eligibility was confirmed. Demographic and clinical information were collected at enrollment.

### Perimetric Devices

#### Reference Device

Standard automated perimeter was performed using the Octopus 900 perimeter, employing the G-pattern and dynamic thresholding strategy with a Goldmann size III stimulus. Gaze tracking was enabled, and background luminance was set to 10 cd/m^2^. All examinations were conducted by an experienced clinical perimetrist.

#### Head-Mounted Device

The VO (PeriVision AG) is a US Food and Drug Administration-cleared and Conformité Européenne-marked head-mounted perimeter built on the Pico Neo 3 VR (Qingdao Pico Technology Co). The device replicates the same G-pattern and dynamic strategy as the reference device, with identical background luminance (10 cd/m^2^). Fixation was monitored using integrated eye tracking combined with Heijl–Krakau blind spot monitoring. Although binocular testing is supported, examinations were performed monocularly.

Due to the limited maximum luminance of the headset (approximately 105 cd/m^2^, compared with 1273 cd/m^2^ for the Octopus 900), the VO applies a proprietary algorithm to compensate for low-contrast limitations. This compensation is achieved by dynamically computing the diameter and RGB color of visual stimuli based on a target intensity in decibels (dB). The algorithm maps intensities (dB) to luminance values (cd/m^2^), interpolates the corresponding RGB values, and scales the stimulus area to ensure perceptual consistency across different luminance levels. This adjustment extends the device’s effective dynamic range and improves sensitivity estimation in regions of advanced field loss. Testing was automatically paused when eye movements exceeded 5° deviation and resumed once fixation was re-established. The number of fixation losses was recorded for each session.

All VO examinations were conducted by a single trained clinician (C.H.).

### Testing Procedure

Each participant completed both perimetry examinations during a single visit. Conventional SAP perimetry was performed first, followed by testing with the VO headset after a short rest period to reduce fatigue. This fixed order was chosen to minimize disruptions to clinical workflows. For participants with bilateral glaucoma, the order of eye testing (right vs. left) was randomized. If only 1 eye met the inclusion criteria, that eye was tested. All tests were conducted in a dimly lit room under standardized lighting conditions.

### Data Processing and Outcome Measures

Corresponding VF locations were manually matched between devices. Analyses focused on pointwise sensitivity thresholds and global indices, including mean sensitivity (MS) and mean defect (MD). For the VO, total deviation (TD) values were computed using the manufacturer’s normative database. Following the conventional SAP device convention, TD at tested VF location was defined as the difference between age-matched normal sensitivity and measured sensitivity at that location.

### Statistical Analysis

Global indices and pointwise values obtained with the VO and the Octopus 900 were compared using complementary statistical approaches assessing association and agreement. Linear associations between devices were quantified using Pearson correlation coefficients (ρ), with corresponding *P* values testing the null hypothesis of no linear association (ρ = 0). To account for measurement error in both devices, Deming regression was additionally performed. The variance δ, which quantifies the relative variability between the 2 measurements, was calculated as the ratio of the variance of Octopus 900 to the variance of VO measurements.

Agreement between devices was evaluated using Bland–Altman analyses, systematic bias, and 95% limits of agreement (LoA). Differences in global indices, test duration, and reliability metrics were assessed using paired statistical tests where appropriate. A 2-sided significance level of *P* < 0.05 was considered statistically significant. Representative VF plots were reviewed qualitatively to illustrate concordance in defect detection.

Simulator-related discomfort was assessed using the Simulator Sickness Questionnaire, a validated self-report instrument designed to quantify symptoms associated with exposure to virtual environments, such as nausea, visual strain, and spatial disorientation.^7^ All analyses were performed using Python version 3.11 (Python Software Foundation).

## Results

### Participant Characteristics

All 73 enrolled participants with glaucoma (120 eyes) completed valid examinations on both devices. The mean age of included participants was 67.0 ± 13.4 years (range 27–90). Based on MD from the Octopus 900, 75 eyes were classified as mild (MD ≤6.0 dB), 26 as moderate (6.0 less than MD ≤11.99 dB), and 19 as severe (MD >11.99 dB). The data included 52 left eyes and 68 right eyes, with 30 participants contributing 1 eye each and 45 participants contributing both.

Mean test duration was comparable between devices, with slightly shorter examinations in the VO (6.3 ± 1.0 min) than the Octopus 900 (6.5 ± 0.8 min; *P* = 0.1). Fixation accuracy during VO was 85.3 ± 18.8%, indicating generally stable fixation performance.

### Correlation Analyses

Global VF indices showed strong agreement between the 2 devices. Mean sensitivity was 21.5 ± 5.9 dB for the VO and 19.1 ± 6.4 dB for the Octopus 900, corresponding to a mean difference (Octopus – VO) of –2.3 ± 2.7 dB. Mean defect values were similarly comparable (VO: 5.3 ± 5.7 dB; Octopus: 6.3 ± 6.2 dB), with a mean difference of 1.0 ± 2.7 dB.

**Figure 1:**
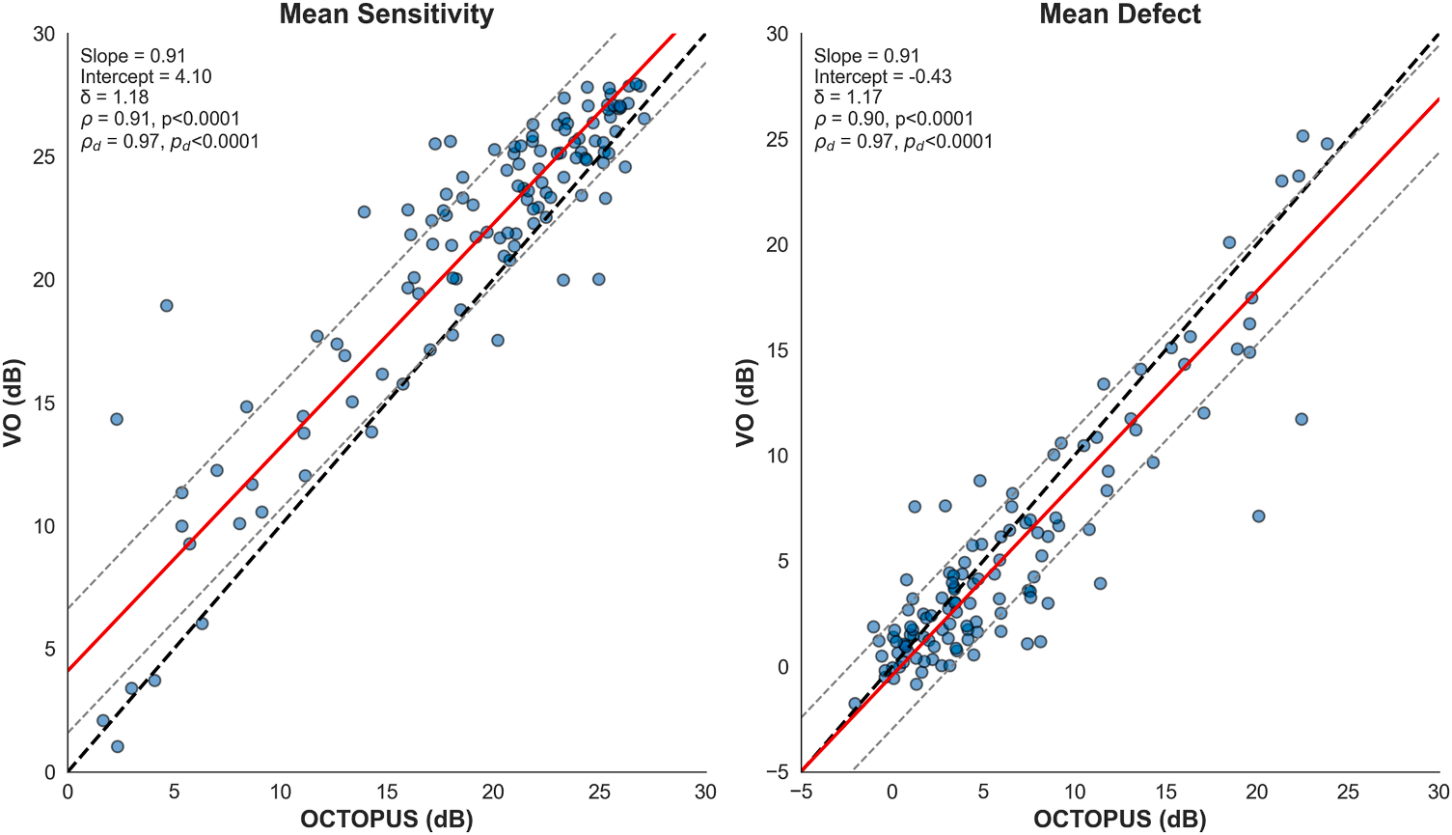
Comparison of visual field global indices between the VisionOne and Octopus 900. Each point represents 1 eye (N = 120). Solid red lines indicate the Deming regression fit. Pearson correlation coefficients demonstrate strong linear associations between devices for mean sensitivity (MS: ρ = 0.91, *P* < 0.0001) and mean defect (MD: ρ = 0.90, *P* < 0.0001). Corresponding Deming regression coefficients were ρ_d_ = 0.97 for MS and MD. dB = decibels; MD = mean defect; MS = mean sensitivity; VO = VisionOne™.

Correlation coefficient demonstrated a very strong linear association between devices (MS: ρ = 0.91, *P* < 0.0001; MD: ρ = 0.90, *P* < 0.0001; Fig 1). Deming regression, accounting for measurement error in both perimeters, confirmed these findings (MS and MD: ρ_d_ = 0.97, *P* < 0.0001). Overall, the VO tended to report slightly higher sensitivity and lower defect values than the Octopus 900; these differences were within the expected test–retest variability of SAP.

**Supplemental Figure S1:**
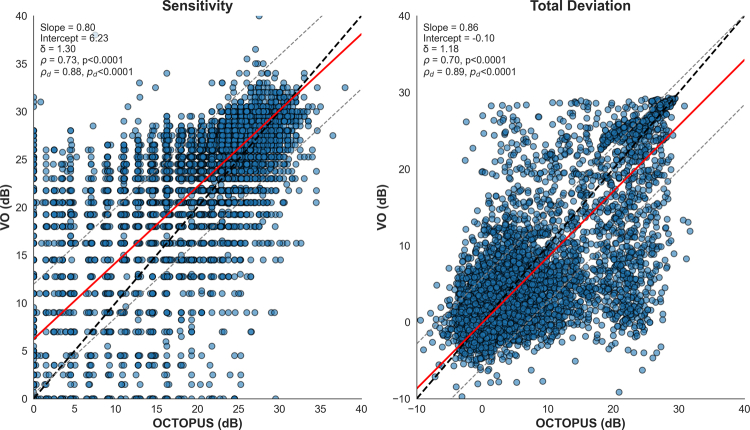


Pointwise sensitivity and TD values also demonstrated a strong linear association between devices (sensitivity: ρ = 0.73, *P* < 0.0001; TD: ρ = 0.70, *P* < 0.0001; Supplementary Figure S1, available at www.ophthalmologyscience.org). Deming regression analyses confirmed these findings, yielding ρ_d_ = 0.88 (*P* < 0.0001) and ρ_d_ = 0.89 (*P* < 0.0001) for sensitivity and TD, respectively.

### Agreement and Bias Analysis

**Figure 2:**
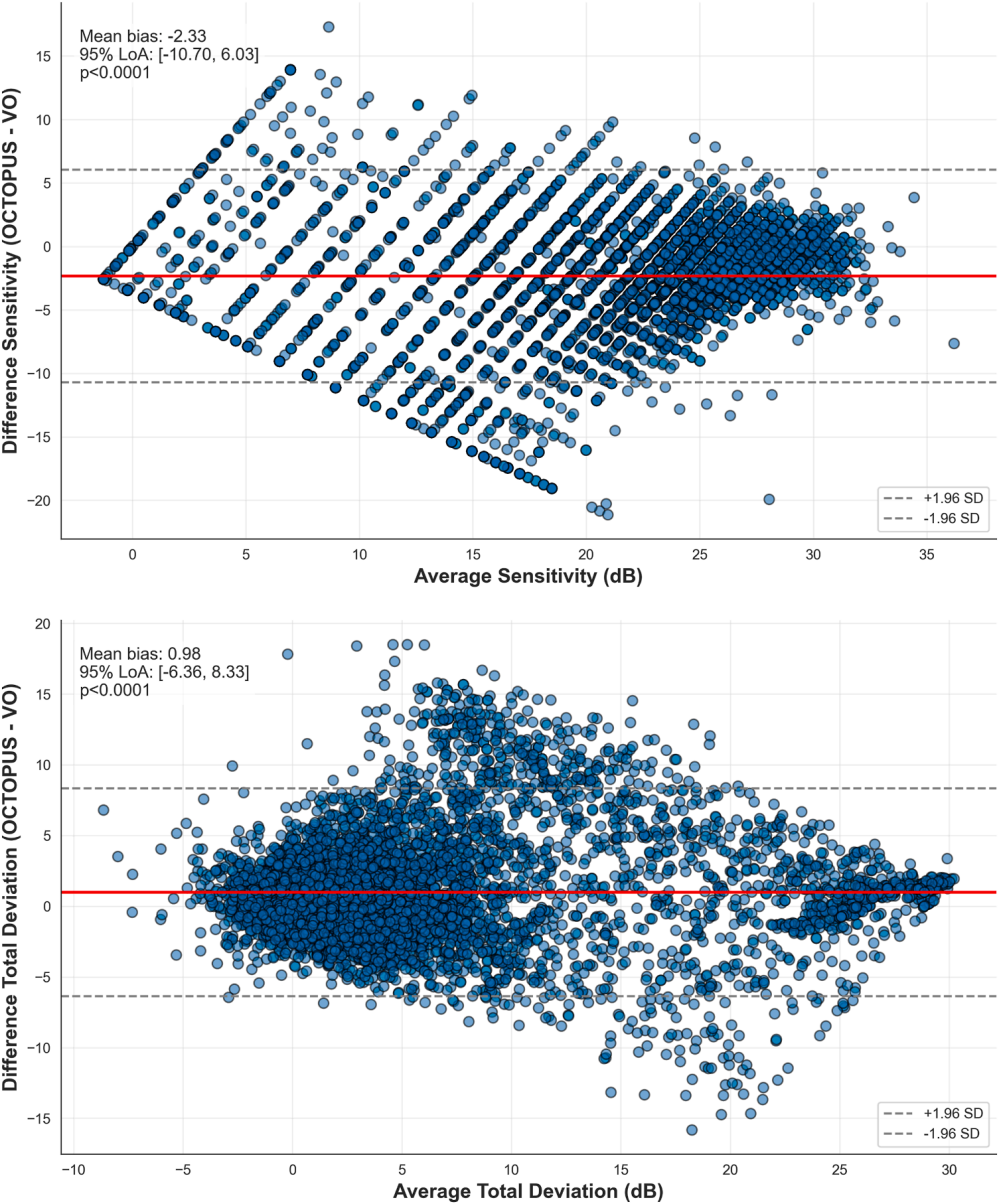
Bland–Altman plot comparing pointwise measurements between the VisionOne and Octopus 900. (Top) Sensitivity: The red line represents the mean bias (–2.33 dB), and the dashed gray lines indicate the 95% limits of agreement (–10.70 to +6.03 dB). (Bottom) Total deviation: The red line represents the mean bias (0.98 dB), and the dashed gray lines indicate the 95% limits of agreement (–6.36 to +8.33 dB). dB = decibels; SD = standard deviation; VO = VisionOne™.

Bland–Altman analyses of pointwise sensitivity values demonstrated good agreement between devices (Fig 2, top). The MS bias was –2.33 dB, indicating a slight overestimation by the VO, with 95% LoA ranging from –10.70 to +6.03 dB (*P* < 0.0001). For TD values, the mean bias was 0.98 dB, with 95% LoA ranging from –6.36 to +8.33 dB (*P* < 0.0001) (Fig 2, bottom). No evidence of proportional bias was observed.

To evaluate performance across the disease spectrum, sensitivity Bland–Altman analyses were stratified by glaucoma severity. As expected, variability increased with greater disease severity, consistent with known SAP test–retest characteristics in advanced disease. In mild glaucoma, the MS bias was –1.68 dB (95% LoA: –8.47 to +5.11 dB, *P* < 0.0001). In moderate glaucoma, the MS bias was –2.94 dB (–12.64 to +6.76 dB, *P* < 0.0001). In the severe glaucoma subgroup, the mean bias was –4.03 dB in severe glaucoma (–14.56 to +6.51 dB, *P* < 0.01).

### Stage-Wise Comparison

**Figure 3:**
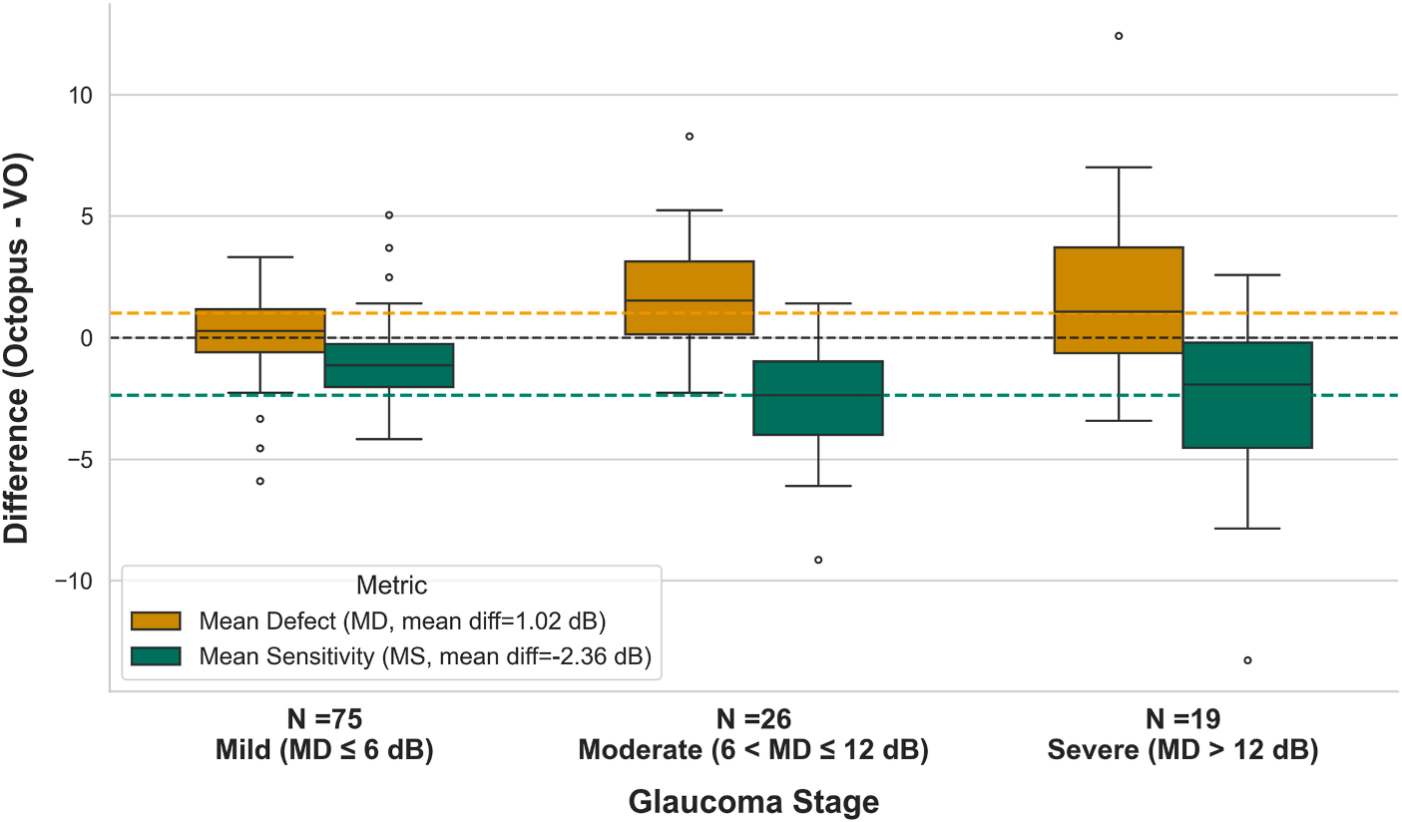
Comparison of mean sensitivity and mean defect differences between the Octopus 900 and VisionOne™ across glaucoma stages. Boxplots represent per-eye differences, with dashed horizontal lines indicating zero difference (black) and the overall mean difference across all eyes (colored). dB = decibels; VO = VisionOne™.

Interdevice differences remained stable across glaucoma severity stages, with mild and moderate glaucoma showing values close to the overall mean (Fig 3). Increased variance with severity falls within the increased test–retest variability typically reported for VF loss.

### Spatial Analysis

**Figure 4:**
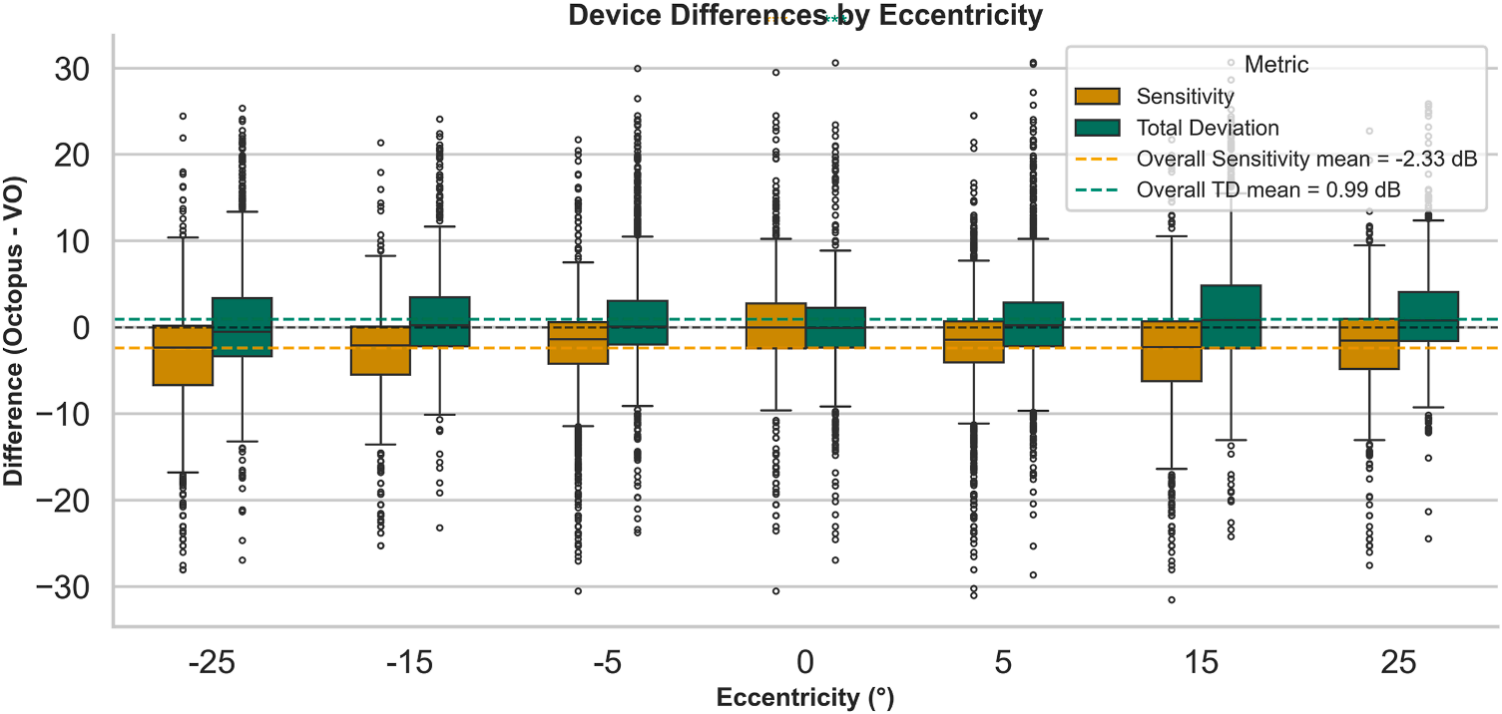
Distribution of differences between the Octopus 900 and VisionOne in sensitivity and total deviation across horizontal visual field eccentricities. Each box represents the spread of values at a given eccentricity. The overall mean difference is shown as dashed lines (orange = sensitivity, green = total deviation, black = no difference). dB = decibels; VO = VisionOne™.

Spatial comparisons showed that interdevice differences did not vary systematically across horizontal VF eccentricities (Fig 4). Both sensitivity and TD values exhibited minor fluctuations with eccentricity, with a trend toward slightly higher sensitivity values measured by the VO in more peripheral locations.

**Supplemental Figure S2:**
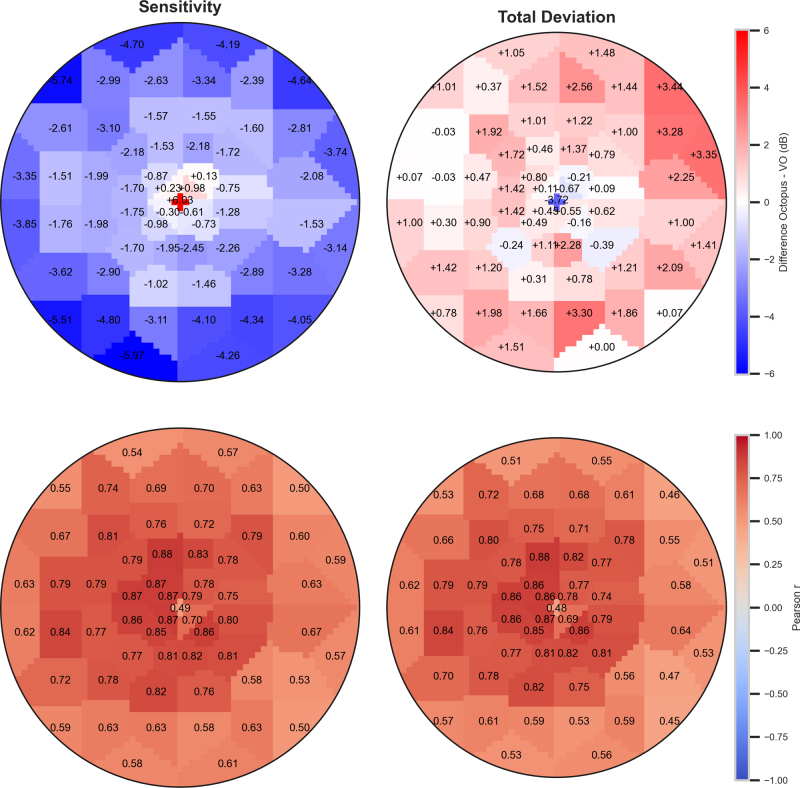


Location-wise correlation maps (Supplementary Figure S2, available at www.ophthalmologyscience.org) demonstrated stronger agreement between devices at central VF locations, with increased variability toward the periphery. This pattern is consistent with established characteristics of perimetric testing, where central test points typically show lower test–retest variability than peripheral locations.^8^

### Reliability Metrics

Reliability indices were comparable between devices. False-positive rates did not differ significantly between the VO and the Octopus 900 (5.5 ± 9.8% vs. 5.7 ± 11.1%, respectively; *P* = 0.9) nor did false-negative rates (8.4 ± 16.1% vs. 6.4 ± 11.4%; *P* = 0.2). These findings indicate similar test quality for VRP and conventional SAP perimetry.

### Qualitative Concordance

**Figure 5:**
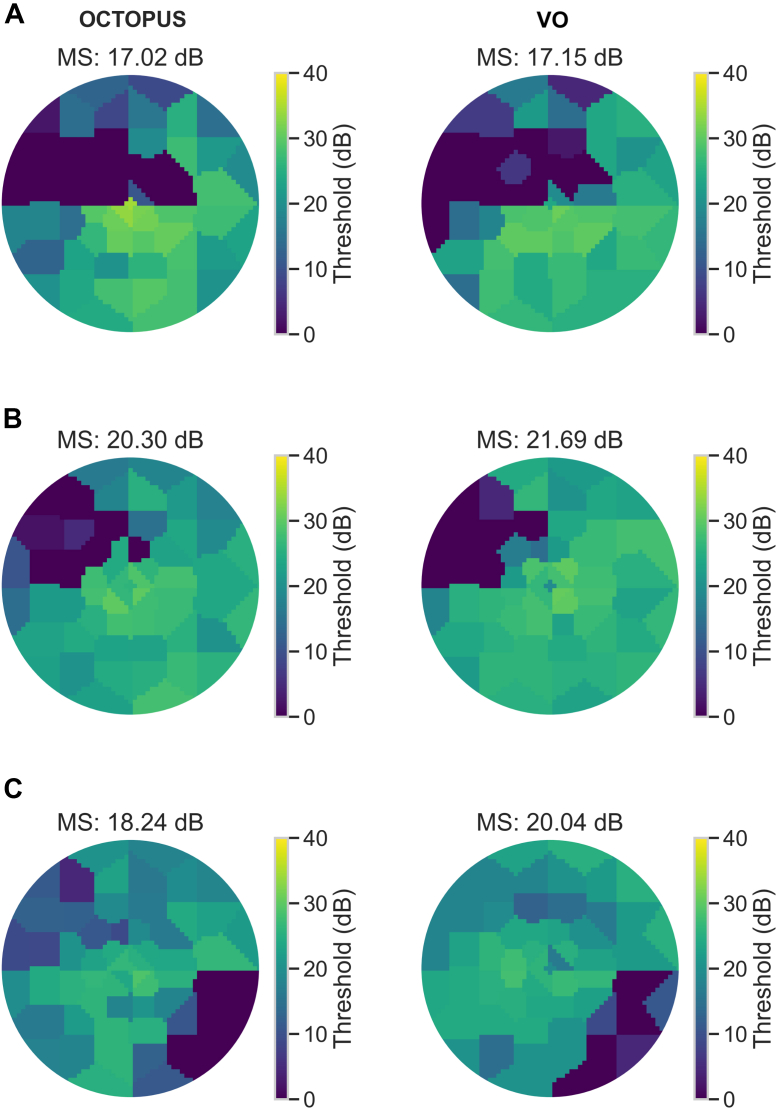
Three examples of glaucomatous visual fields tested with the Octopus 900 (left) and VisionOne™ (VO, right). Each row (**A–C**) shows matched tests using the G-pattern, with colors representing sensitivity thresholds (lower values = brighter stimuli). dB = decibels; MS = mean sensitivity

Qualitative inspection of representative glaucomatous VFs shown in Figure 5 demonstrated high concordance between devices. The VO reproduced both MS and localized defect patterns observed with the Octopus 900, including arcuate defects, nasal steps, and subtle depressions, with comparable depth and spatial extent.

### Simulator Sickness

Simulator-related discomfort was evaluated using the 16-item Simulator Sickness Questionnaire, with symptom severity graded as "none," "slight," "moderate," or "severe." Four participants did not complete the questionnaire, yielding a total of 1104 individual item responses. Overall, simulator-related discomfort was low, and no participants discontinued testing due to adverse effects.

**Figure 6:**
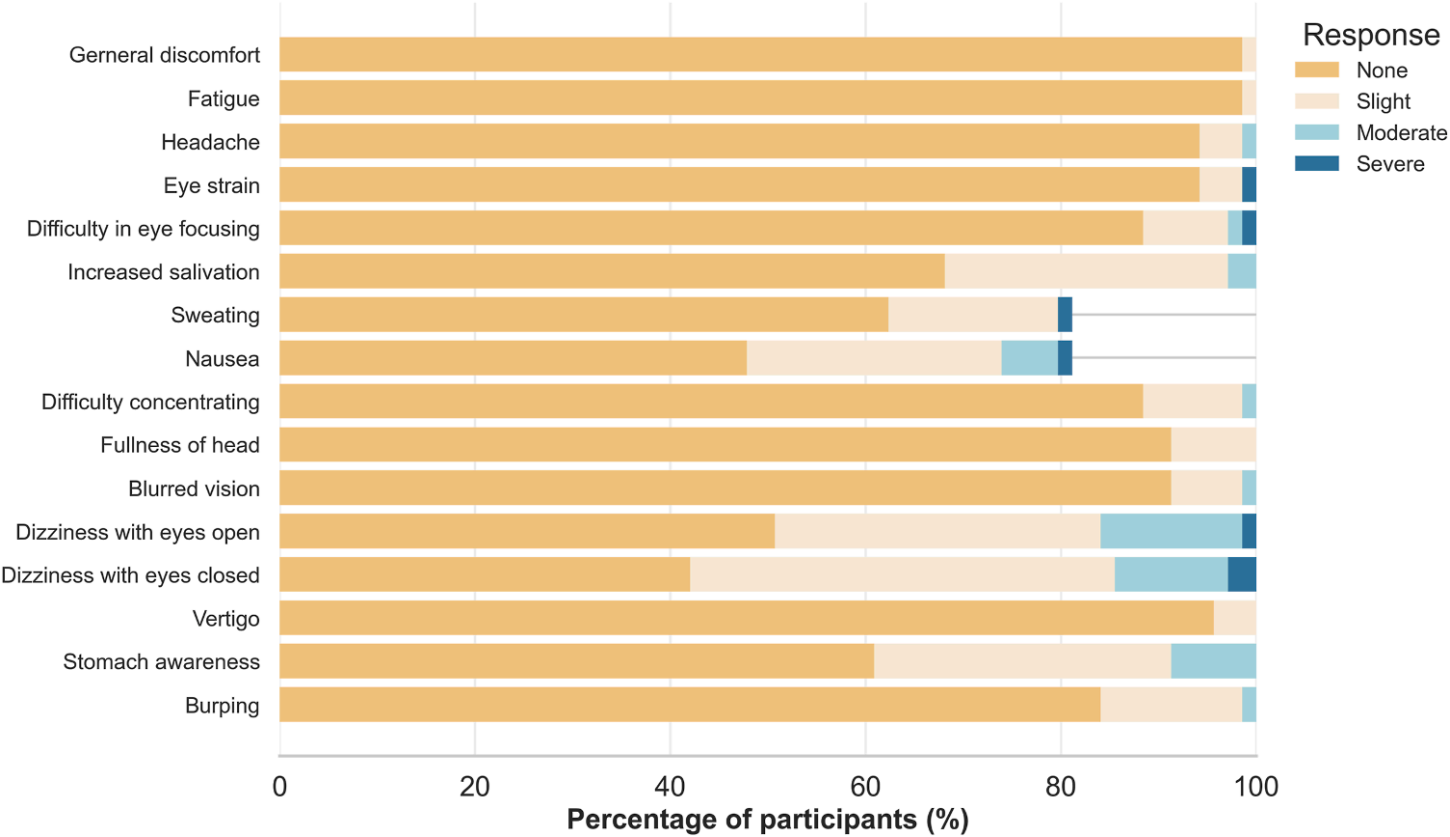
Item-level distribution of Simulator Sickness Questionnaire responses. Horizontal stacked bars show the percentage of participants reporting each severity level (“none,” “slight,” “moderate,” and “severe”) for 16 Simulator Sickness Questionnaire items, including general discomfort, oculomotor- and disorientation-related symptoms.

Item-level analysis (Fig 6) showed that most responses were rated as “none” (78.53%) or “slight” (15.31%) symptoms. “Moderate” (3.17%) or “severe” (0.63%) symptoms occurred infrequently. Oculomotor symptoms (e.g., eye strain, blurred vision, and difficulty focusing) were the most frequently reported adverse events; however, the majority were rated as “slight” and only 14.49% reporting "moderate" to "severe" (reported by 2 participants) visual strain. Spatial disorientation was minimal, with 3 participants reporting "moderate" to “severe” dizziness (with or without eyes open), and 1 participant reporting “moderate” vertigo. Detailed item-level frequency distributions are presented in Figure 6 (some participants provided incomplete responses to specific items, accounting for cumulative bars that do not reach 100%).

## Discussion

In this study, we evaluated the performance of a commercially available, regulatory-approved head-mounted VRP (VisionOne) against a conventional SAP (Octopus 900) in individuals with glaucoma with a wide range of disease severity.

Global VF indices showed strong linear associations between the VO and the Octopus 900, with high Pearson correlation coefficients for both MS and MD. Deming regression, accounting for measurement error in both perimeters, confirmed these findings. Bland–Altman analyses revealed a small systematic bias, with the VO tending to report slightly higher sensitivity and lower defect values. Importantly, the magnitude of this bias was within the known test–retest variability of SAP,^8^ and no proportional bias was observed, indicating stable interdevice differences across the measurement range. Interestingly, when using the respective device-specific normative databases to evaluate TD values, this systematic bias was substantially reduced. This demonstrates that software-based normative databases effectively neutralize hardware-dependent display differences, yielding near-equivalent clinical metrics.

Given the absence of direct interdevice benchmarking data comparing VRP and SAP, utilizing known SAP intradevice test–retest variability provides the most rigorous available baseline for comparison. In this context, the 95% LoA for the overall pointwise sensitivity range is encouraging. For clinical perspective, SAP testing itself exhibits a notable intrinsic, native test–retest variability of approximately –8.0 to ∼+8.0 dB even when deploying identical perimetric testing strategies.^9^ Furthermore, our observed range of variability is entirely consistent with recent VRP validation literature against SAP.^10^ Nevertheless, to further isolate platform-specific repeatability, a dedicated clinical trial is planned to explicitly characterize the test-retest variability of the VRP.

At the pointwise level, sensitivity and TD values demonstrated strong correlations between devices, indicating consistent spatial correspondence across individual VF locations. As expected, correlation coefficients were higher centrally and decreased toward the periphery, reflecting the increased variability of perimetric measurements at larger eccentricities. Despite this expected peripheral noise, the spatial configuration and depth of localized glaucomatous defects were reliably reproduced by the VO platform, supporting its capacity to track localized regional loss.

Notably, individuals with mild glaucoma are frequently overrepresented in validation studies,^3^ which introduces spectrum bias and limits the ability to draw firm conclusions regarding advanced disease. By evaluating a cohort that includes 19 eyes with severe glaucoma, our study explicitly addresses this gap in the literature. Interestingly, while the systematic review found that most VRP systems tend to underestimate sensitivity and defect size in patients with glaucoma, the VO demonstrated a slight overestimation of sensitivity overall and across all severity stages. Despite this directional difference, our stage-wise analysis revealed that agreement between the VO and Octopus devices remained stable across the disease spectrum. The increased variance observed in advanced stages aligns with the higher test–retest variability typically reported in conventional perimetry for severe VF loss.

Reliability indices and test duration were comparable between devices, with no significant differences in false-positive or false-negative rates. Discomfort related to the simulator was generally low to moderate, with no participants discontinuing the testing process. The symptoms experienced were mainly oculomotor, while nausea and disorientation occurred infrequently. This suggests that head-mounted perimetry is a feasible option for populations with glaucoma.

Although the VRP system allows for head movement, the testing approach is predominantly static and focused on fixation, which minimizes engagement with self-motion or spatial updating processes. This likely limits the involvement of the vestibular system, helping to explain the low incidence of motion sickness symptoms typically reported in immersive VR environments.^6^

The portability and reduced infrastructure requirements of head-mounted perimetry may facilitate decentralized glaucoma care, including community screening and home monitoring. Our findings demonstrate that a commercially available VR-based perimeter can provide VF measurements comparable to those obtained with conventional SAP under controlled conditions. However, this study has limitations. The testing order was not randomized, which might have introduced fatigue effects, even though participants had prior perimetry experience and were given rest periods. Additionally, the inherent variability at peripheral test locations is a limitation that all perimetric techniques share. Longitudinal studies are necessary to evaluate test–retest variability and to detect progression with head-mounted perimetry.

The VO head-mounted VRP demonstrated a strong association and good agreement with conventional SAP for both global and pointwise VF measures in glaucoma. Reliability, test duration, and user tolerance were comparable between devices, supporting the clinical validity of immersive head-mounted perimetry as an alternative platform for VF assessment.

## Declaration of Generative AI and AI-Assisted Technologies in the Writing Process

During the preparation of this work, the author(s) used Grammarly (Grammarly Inc., San Francisco, CA, USA) and DeepL Translator (DeepL SE, Cologne, Germany) in order to edit language and assist translation during manuscript preparation. After using this tool/service, the author(s) reviewed and edited the content as needed and take(s) full responsibility for the content of the publication.

## Supplementary Data

Supplemental Figure captions
