# Supplementary material for: Evaluation of a Head-Mounted Virtual Reality Perimeter Compared with Stationary Perimeters across All Stages of Glaucoma
Source: Ophthalmol Sci. 2026 Jul 4;6(9):101314. doi: 10.1016/j.xops.2026.101314 (PMC13475070; doi:10.1016/j.xops.2026.101314)
Supplement: Supplementary file 1 [file mmc1.pdf]

APPENDIX: SUPPLEMENTAL MATERIAL

Supplementary Figures

Supplementary Figure S1. Comparison of pointwise sensitivity and total deviation values between the VisionOne™ (VO) and Octopus 900. Each point represents a single visual field test location. Solid red lines indicate the Deming regression fit. Pearson correlation coefficients demonstrate strong linear associations between devices for sensitivity ($\rho = 0.73$, $p < 0.0001$) and total deviation ($\rho = 0.70$, $p < 0.0001$). Corresponding Deming regression coefficients were $\rho_d = 0.88$ and $\rho_d = 0.89$ for sensitivity and total deviation, respectively.

Supplementary Figure S2. Spatial comparison of visual field measurements between the Octopus 900 and VisionOne™ (VO). Top row: Mean differences (Octopus – VO) for sensitivity (left) and total deviation (right), shown as Voronoi maps. Blue areas indicate higher VO values, red areas higher Octopus values. Bottom row: Pearson correlation coefficients at each test location for sensitivity (left) and total deviation (right). Warmer colors represent stronger correlations (range –1 to +1). Central test locations showed the highest agreement, consistent with known perimetric reliability patterns.
